# Evaluation and updating of the Medical Malacology Collection (Fiocruz-CMM) using molecular taxonomy

**DOI:** 10.1186/2193-1801-3-446

**Published:** 2014-08-20

**Authors:** Cryslaine Aguiar-Silva, Cristiane Lafetá Furtado Mendonça, Pedro Henrique da Cunha Kellis Pinheiro, Silvia Gonçalves Mesquita, Omar dos Santos Carvalho, Roberta Lima Caldeira

**Affiliations:** Laboratório de Helmintologia e Malacologia Médica do Centro de Pesquisas René Rachou-Fiocruz, Av. Augusto de Lima 1715, Belo Horizonte, MG 30190-002 Brasil; Pontifícia Universidade Católica de Minas Gerais, Belo Horizonte, MG Brasil

**Keywords:** Zoological collection, *Biomphalaria*, PCR-RFLP, Morphological identification, Molecular taxonomy

## Abstract

**Background:**

The Medical Malacology Collection (Coleção de Malacologia Médica, Fiocruz-CMM) is a depository of medically relevant mollusks, especially from the genus *Biomphalaria*, which includes the hosts of *Schistosoma mansoni*. Taxonomic studies of these snails have traditionally focused on the morphology of the reproductive system. However, determination of some species is complicated by the similarity shown by these characters. Molecular techniques have been used to try to overcome this problem.

**Description:**

The Fiocruz-CMM utilizes morphological and/or molecular method for species’ identification. However, part of the collection has not been identified by molecular techniques and some points were unidentified. The present study employs polymerase chain reaction-based analysis of restriction fragment length polymorphisms (PCR-RFLP) to evaluate the identification of *Biomphalaria* in the Fiocruz-CMM, correct existing errors, assess the suitability of taxonomic synonyms, and identify unknown specimens. The results indicated that 56.7% of the mollusk specimens were correctly identified, 4.0% were wrongly identified, and 0.4% was identified under taxonomic synonyms. Additionally, the PCR-RFLP analysis identified for the first time 17.6% of the specimens in the Collection. However, 3.1% of the specimens could not be identified because the mollusk tissues were degraded, and 18.2% of the specimens were inconclusively identified, demonstrating the need for new taxonomic studies in this group.

**Conclusion:**

The data was utilized to update data of Environmental Information Reference Center (CRIA). These studies demonstrate the importance of using more than one technique in taxonomic confirmation and the good preservation of specimens’ collection.

## Introduction

The Medical Malacology Collection (Coleção de Malacologia Médica, Fiocruz-CMM) located in the Medical Helminthology and Malacology Laboratory (Laboratório de Helmintologia e Malacologia Médica, LHMM) of the René Rachou Research Center (Centro de Pesquisas René Rachou, CPqRR/Fiocruz) comprises approximately 12,000 mollusk specimens with medical or veterinary relevance. The collection consists mainly of representatives of the genus *Biomphalaria* (Preston, 1910). This genus includes the intermediate hosts of the trematode *Schistosoma mansoni* Sambon, 1907, a causative agent of intestinal schistosomiasis.

This collection was initiated in 1993, when the LHMM began to receive mollusk specimens from various locations for morphological species identification and evaluation of trematode infection. The Fiocruz-CMM has specimens from Germany, France, Argentina, Bolivia, Brazil, Chile, Colombia, Costa Rica, Cuba, Ecuador, Mexico, Paraguay, Dominican Republic, Uruguay and Venezuela. Upon arriving at LHMM, mollusks of the genus *Biomphalaria* are examined to verify the presence of *S. mansoni* cercariae, and five samples of each collection point are anesthetized and sacrificed. Their shells and body are separated, and fragments from the cephalopodal region are cryopreserved (for molecular studies). This material is later encoded and routed to Fiocruz-CMM.

Classical species identification involves on comparison of morphological characters of the shell (such as diameter, width and number of whorls), and male and female reproductive organs, as described by Paraense (1975, 1981, 1984, 1988, 1990), Paraense and Deslandes (1958a, 1958b), Paraense et al. (1992), and Estrada et al. (2005). However, identification can be hindered by interspecific morphological similarities in these characters, the small size of some specimens, and inadequate processes (Paraense 1975; Caldeira et al. 1998; Spatz et al. 1999). During the early 2000’s, therefore, the LHMM began to use molecular tools to aid in the identification of these mollusks. Currently, polymerase chain reaction-based analysis of restriction fragment length polymorphisms (PCR-RFLP) within the internal transcribed spacer (ITS) of the ribosomal RNA gene is used to distinguish *Biomphalaria* species, as defined by Vidigal et al. (2000a) and Teodoro et al. (2010).

However, part of the mollusks of the Fiocruz-CMM collection has not been characterized using molecular techniques, and some deposited specimens remain unidentified. Thus, the objective of this study was to evaluate and update the identification of *Biomphalaria* specimens in the Fiocruz-CMM collection using molecular taxonomic techniques. Unidentified specimens were analyzed using morphological and molecular techniques; specimens that had been identified using only morphological traits were confirmed or corrected; and the resulting taxonomic data were updated and made available to users of the collection.

## Methodology

### Specimen selection

Collection points deposited between 1993 and 2011 that included *Biomphalaria* specimens were selected. There were 1,398 such collection points. Specimens from 198 of these collection points had already been identified by PCR-RFLP and were not included in this study. Thus, at least one specimen from each of 1,200 collection points was analyzed using morphological traits and/or PCR-RFLP.

### Morphological identification

The mollusks were dissected according to the procedures of Deslandes (1951) and identified based on the morphology of their shells, reproductive and excretory organs (Paraense 1966a, 1975, 1981, 1984, 1988, 1990; Paraense and Deslandes 1958a, 1958b; Estrada et al. 2005). The dissected specimens were returned to the collection for further morphological studies.

### Molecular techniques

From the samples selected for molecular identification, the fragments from the cephalopodal region that were cryopreserved were divided into two parts. One part was retained in cryopreservation, and the other part was used for DNA extraction. Total DNA was extracted using the Wizard kit (Promega) according to the manufacturer’s instructions. The PCR-RFLP technique was performed according to the methods of Vidigal et al. (1998). For species identification, the resulting profiles were compared to those obtained by Spatz et al. (1999), Vidigal et al. (2000a), Caldeira et al. (2000), Vidigal et al. (2001) and Teodoro et al. (2010).

### Analysis and update of the collection database

The specimens were grouped into six categories: 1) correctly identified, 2) erroneously identified, 3) identified under taxonomic synonyms, 4) inconclusively identified, 5) unidentified due to tissue degradation, and 6) newly identified (specimens that had been deposited unidentified).

## Results

### Selected mollusks

The Fiocruz-CMM collection included 20 *Biomphalaria* morphotypes obtained from 1,200 collection points (Table 1).

**Table 1:** **Number (and percentage) of collection points with identified specimens of each** ***Biomphalaria*** **morphotype before (initial) and after this study (final)**

Morphotype	Initial collection points (%)	Final collection points (%)
*Biomphalaria amazonica* Paraense 1966a	9 (0.7)	7 (0.6)
*Biomphalaria cousini* Paraense 1966b	4 (0.3)	4 (0.3)
*Biomphalaria edisoni* Estrada et al., 2006	2 (0.2)	2 (0.2)
*Biomphalaria glabrata* (Say, 1818)	331 (27.6)	382 (31.8)
*Biomphalaria havanensis* (Pfeiffer, 1839)	6 (0.5)	20 (1.7)
*Biomphalaria intermedia* Paraense & Deslandes, 1962	21 (1.75)	40 (3.3)
*Biomphalaria kuhniana* (Clessin, 1883)	19 (1.6)	23 (1.9)
*Biomphalaria obstructa* (Morelet, 1849)^a^	4 (0.3)	-
*Biomphalaria occidentalis* Paraense, 1981	23 (1.9)	30 (2.5)
*Biomphalaria oligoza* Paraense, 1974	3 (0.2)	10 (0.8)
*Biomphalaria orbignyi* Paraense, 1975	-	1 (0.1)
*Biomphalaria peregrina* (Orbigny, 1835)	116 (9.7)	208 (17.3)
*Biomphalaria prona* (Martens, 1873)	6 (0.5)	6 (0.5)
*Biomphalaria schrammi* (Crosse, 1864)	14 (1.2)	20 (1.7)
*Biomphalaria* aff. *straminea* ^b^	-	4 (0.3)
*Biomphalaria straminea* (Dunker, 1848)	198 (16.5)	266 (22.2)
*Biomphalaria tenagophila* (Argentina)^c^	-	6 (0.5)
*Biomphalaria tenagophila* (Orbigny, 1835)	96 (8)	111 (9.2)
*Biomphalaria temascalensis* (Rangel-Ruiz, 1987)^a^	1 (0.1)	-
*Biomphalaria tenagophila guaibensis* Paraense, 1984	14 (1.2)	23 (1.9)
Unidentified	333 (27.8)	37 (3.1)
**Total**	1,200 (100)	1,200 (100)

^a^Junior synonym of *Biomphalaria havanensis*.

^b^Specimens morphologically similar to *Biomphalaria straminea* and originating from Espinillar, Uruguay, the type locality of *Biomphalaria* aff. *straminea*.

^c^Specimens morphologically identical to *Biomphalaria tenagophila* but with a distinct molecular profile.

### Taxonomic analyses

Among the 1,200 analyzed collection points, the species identifications were 1) correct for 56.7% of the specimens, 2) erroneous for 4.0%, 3) synonymous for 0.4% (these specimens were identified as *B. obstructa* and *B. temascalensis*, junior synonyms of *B. havanensis*), 4) inconclusive for 18.2%, 5) undetermined due to tissue degradation for 3.1%, and 6) newly determined for 17.6%, including one specimen of *B. orbignyi*. The inconclusively identified and degraded specimens were retained in the collection for further evaluation.

The confirmed, corrected, synonymized and newly determined species identifications were updated in the collection registry and in the Environmental Information Reference Center (Centro de Referência em Informação Ambiental, CRIA) database (http://splink.cria.org.br).

Table 2 lists the erroneous species identifications that were found in the Fiocruz-CMM collection. Nearly half of the erroneous identifications were originally identified as *B.tenagophila.*

**Table 2:** **Erroneous species identifications among the analyzed collection points**

Previous identification	Current identification	No. of collection points with each error
*Biomphalaria tenagophila*	*Biomphalaria peregrina*	10
*Biomphalaria tenagophila*	*Biomphalaria glabrata*	8
*Biomphalaria straminea*	*Biomphalaria kuhniana*	5
*Biomphalaria peregrina*	*Biomphalaria tenagophila*	4
*Biomphalaria amazonica*	*Biomphalaria cousini*	2
*Biomphalaria tenagophila*	*Biomphalaria occidentalis*	2
*Biomphalaria glabrata*	*Biomphalaria tenagophila*	2
*Biomphalaria glabrata*	*Biomphalaria straminea*	2
*Biomphalaria intermedia*	*Biomphalaria peregrina*	1
*Biomphalaria intermedia*	*Biomphalaria straminea*	1
*Biomphalaria occidentalis*	*Biomphalaria tenagophila*	1
*Biomphalaria peregrina*	*Biomphalaria intermedia*	1
*Biomphalaria peregrina*	*Biomphalaria straminea*	1
*Biomphalaria prona*	*Biomphalaria kuhniana*	1
*Biomphalaria schrammi*	*Biomphalaria peregrina*	1
*Biomphalaria straminea*	*Biomphalaria intermedia*	1
*Biomphalaria straminea*	*Biomphalaria glabrata*	1
*Biomphalaria straminea*	*Biomphalaria occidentalis*	1
*Biomphalaria straminea*	*Biomphalaria oligoza*	1
*Biomphalaria tenagophila*	*Biomphalaria straminea*	1
*Biomphalaria tenagophila*	*Biomphalaria tenagophila guaibensis*	1
**Total number of collection points with errors**		48

**Figure 1:**
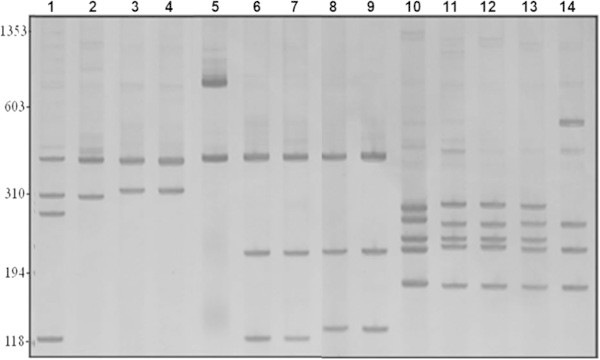
***Biomphalaria*** **restriction profiles: 6% polyacrylamide gel showing the PCR-RFLP profiles obtained by digesting the rDNA ITS region of** ***Biomphalaria*** **mollusks with** ***Dde*** **I (lanes 1-9) and** ***Alu*** **I (lanes 10-14).** Lane 1: *Biomphalaria straminea* (Minas Gerais, Brazil); 2: *Biomphalaria intermedia* (Minas Gerais, Brazil); 3: *Biomphalaria straminea* (Corrientes, Argentina); 4: *Biomphalaria straminea* (Espinillar, Uruguay); 5: *Biomphalaria tenagophila* (Minas Gerais, Brazil); 6-7: *Biomphalaria tenagophila* (Corrientes, Argentina); 8: *Biomphalaria tenagophila guaibensis* (Rio Grande do Sul, Brazil); 9: *Biomphalaria occidentalis* (Minas Gerais, Brazil); 10: *Biomphalaria tenagophila* (Minas Gerais, Brazil); 11-12: *Biomphalaria tenagophila* (Corrientes, Argentina); 13: *Biomphalaria tenagophila guaibensis* (Rio Grande do Sul, Brazil); 14: *Biomphalaria occidentalis* (Minas Gerais, Brazil). Values to the left correspond to molecular weights in base pairs (bp).

**Figure 2:**
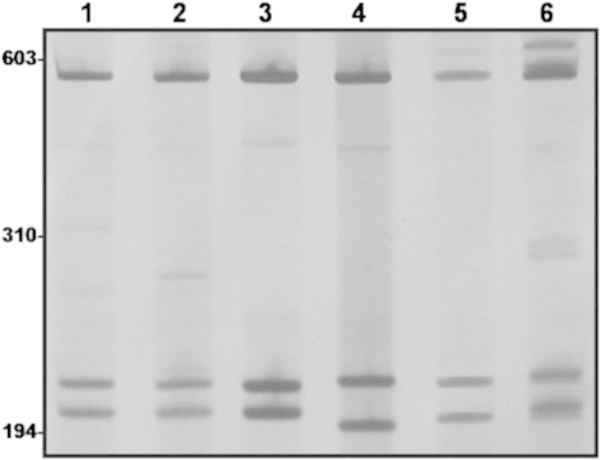
**Restriction profiles of** ***Biomphalaria peregrina*** **and** ***Biomphalaria oligoza.*** Lanes 1-2: a specimen with the morphology of *Biomphalaria peregrina* and the molecular profile of *Biomphalaria oligoza*; 3: a specimen with the morphology and molecular profile of *Biomphalaria oligoza*; 4: a specimen with the morphology and molecular profile of *Biomphalaria peregrina*; 5-6: a specimen with the morphology of *Biomphalaria peregrina* and a molecular profile that is intermediate between the two species. Values to the left correspond to molecular weights in base pairs (bp).

Three groups were prominent among the inconclusively identified specimens. 1) Specimens from the provinces of Corrientes, Argentina and Espinillar, Uruguay (four collection points) were morphologically similar to *B. straminea*, but their restriction profile for the enzyme *Dde*I differed from that of *B. straminea* (which had been previously established), here represented by sample from Minas Gerais, Brazil (Figure 1). 2) Specimens from the provinces of Corrientes, Argentina and Espinillar, Uruguay (six collection points) were morphologically similar to *B. tenagophila* from Brazil, but their restriction profiles for the enzymes *Dde*I and *Alu*I were similar to those of *B. t. guaibensis* from Rio Grande do Sul, Brazil (Figure 1). 3) Specimens from 208 collection points in various Brazilian states were morphologically identified as *B. peregrina*, but although some of these specimens had molecular profiles characteristic of that species, others had the molecular profile of *B. oligoza* (which had been previously established), here represented by sample from Rio Grande do Sul, Brazil (Figure 2). In an attempt to clarify the identity of these specimens, a portion of the mitochondrial cytochrome c oxidase subunit I (COI) gene was amplified by PCR, and the restriction enzymes *Cla*I, *Rsa*I, and *Alu*I were used to generate PCR-RFLP profiles. There was no restriction site for the enzymes *Cla*I and *Rsa*I. For *Alu*I, the specimens morphologically identified as *B. peregrina* sometimes showed *B. peregrina* profiles and sometimes showed *B. oligoza* profiles (data not shown).

## Discussion

The Fiocruz-CMM collection contains taxonomic groups of medical or veterinary relevance to aid in the control of schistosomiasis, support research, and contribute to human-resources development. Considering the global mobilization, consolidation, institutionalization, and organization of biological collections, studies evaluating the taxonomic accuracy of such collections are broadly relevant (Godfray and Knapp 2004; Egler and Santos 2006). The morphological identification of *Biomphalaria* species is challenging (Paraense 1988; Paraense et al. 1992), and PCR-RFLP has been used to aid in species identification (Spatz et al. 1999; Vidigal et al. 2000a; Caldeira et al. 2000; Carvalho et al. 2004). Thus, both tools were used to the taxonomic accuracy of the Fiocruz-CMM collection.

Initially, 8,831 specimens from 1,398 collection points were selected for this study. Specimens from 333 collection points were unidentified, while specimens from 198 collection points had already received molecular confirmation. Thus, this study evaluated the species identifications of mollusks from 1,200 collection points deposited at Fiocruz-CMM.

Identification errors occurred mainly between *B. peregrina* and *B. tenagophila* (29.1%), due to the similarity of these species’ shell and reproductive organs (Paraense 1966a); and between *B. tenagophila* and *B. glabrata* (20.8%), due to the many identical traits of juvenile and sometimes adults *B. glabrata* and *B. tenagophila* specimens (Barbosa 1964, Paraense and Deslandes 1959). The frequencies of these errors reflect the proportions of these species in the Fiocruz-CMM. The identification of certain *B. havanensis* specimens as *B. temascalensis* and *B. obstructa* was adequate because Yong et al. (2001) and DeJong et al. (2001) have concluded that these three entities actually form a single species, making *B. temascalensis* and *B. obstructa* junior synonyms of *B. havanensis*.

Three groups were considered inconclusive: *B.* aff. *straminea*, *B. tenagophila* from Argentina, and *B. peregrina*. Paraense and Corrêa (1989) classified a population from Espinillar, Uruguay, as *B.* aff. *straminea* due to its similarity to *B. straminea*. Vidigal et al. (1998) obtained molecular profiles with two bands (470 and 310 bp) for *B. straminea* populations from the city of San Miguel and the provinces of Chaco and Corrientes, Argentina, and four bands (470, 310, 280, and 120 bp) for *B. straminea* populations from Brazil. In the present study, specimens from Corrientes, Argentina and Espinillar, Uruguay had profiles with two bands, agreeing with the observations of Vidigal et al. (1998), and were morphologically similar to *B. straminea*.

Specimens from Argentina that were morphologically identified as *B. tenagophila* showed a profile with three bands, similar to that reported by Vidigal et al. (1998) for populations from Chaco and Corrientes, Argentina, that were morphologically identified as *B. tenagophila*. However, LHMM has previously used a profile with two bands to characterize *B. tenagophila* (Vidigal et al. 2000a). Spatz et al. (1999) has observed that *B. tenagophila* from Argentina exhibits greater phylogenetic proximity to both *B. occidentalis* and *B. t. guaibensis* than to *B. tenagophila* from Brazil. Paraense (1961) found no morphological differences between *B. tenagophila* populations from Corrientes, Argentina (the type locality of the species) and Brazil. Paraense (1961) also confirmed that these populations were conspecific by performing controlled crosses.

Some specimens that were morphologically identified as *B. peregrina* showed the molecular profile of that species, while others showed that of *B. oligoza* when using *Alu*I to digest either ITS (Figure 2) or COI (data not shown). The morphological separation of *B. peregrina* and *B. oligoza* is primarily based on the number of prostatic diverticula, which ranges from zero to seven in *B. oligoza* (Paraense 1975) and from eight to twenty-two in *B. peregrina* (Paraense 1966b, 1975). However, the specimens that exhibited the molecular profile of *B. oligoza* in this study had 10 to 18 diverticula. According to Vidigal et al. (2000b), the phylogenetic positions of these two species are uncertain because *B. oligoza* specimens are always grouped together, but *B. peregrina* specimens are ultimately grouped with *B. oligoza*. Thus, the number of diverticula may not be a definitive trait for the separation of these species.

Further morphological and molecular studies and experimental crosses are needed to establish the phylogenetic relationships among the members of these three groups, specially the specimens from inconclusive group. The part of the Cytochrome c oxidase subunit I gene (cox1) will be sequenced to better understanding of the taxonomic status of these species. This region has been widely used in taxonomic studies an approach termed DNA barcode (Hebert et al. 2003).

Some specimens could not be identified due to tissue degradation resulting from the lack of periodic maintenance. This result highlights the importance of adequately maintaining biological collections (Egler and Santos 2006).

The results of this study confirm the relevance of molecular taxonomic techniques in evaluating and updating the species identifications of *Biomphalaria* specimens and the need to guarantee proper specimen preservation. Importantly, the consolidation of this collection and the performance of this study were made possible by funding from Fiocruz and the Minas Gerais Research Foundation (Fundação de Amparo à Pesquisa do Estado de Minas Gerais, Fapemig), confirming the need for financial support to strengthen biological collections.
